# Dimensions of Religiousness and Cancer Screening Behaviors Among Church-Going Latinas

**DOI:** 10.1007/s10943-012-9606-9

**Published:** 2012-05-23

**Authors:** Jennifer D. Allen, John E. Pérez, Claudia R. Pischke, Laura S. Tom, Alan Juarez, Hosffman Ospino, Elizabeth Gonzalez-Suarez

**Affiliations:** 1 Center for Community-Based Research, Dana-Farber Cancer Institute, 450 Brookline Avenue, Boston, MA 02215 USA; 2 Department of Psychology, University of San Francisco, 2130 Fulton Street, San Francisco, CA 94117 USA; 3 Institute for Epidemiology and Prevention Research (BIPS), Achterstr. 30, 28359 Bremen, Germany; 4 Iglesia Cristiana Nueva Vida, 70 White Street, Boston, MA 02128 USA; 5 School of Theology and Ministry, Boston College, 140 Commonwealth Avenue, Chestnut Hill, MA 02467 USA

**Keywords:** Latino, Religion, Spirituality, Cancer screening, Coping, Health

## Abstract

Churches are a promising setting through which to reach Latinas with cancer control efforts. A better understanding of the dimensions of religiousness that impact health behaviors could inform efforts to tailor cancer control programs for this setting. The purpose of this study was to explore relationships between dimensions of religiousness with adherence to cancer screening recommendations among church-going Latinas. Female Spanish-speaking members, aged 18 and older from a Baptist church in Boston, Massachusetts (*N* = 78), were interviewed about cancer screening behaviors and dimensions of religiousness. We examined adherence to individual cancer screening tests (mammography, Pap test, and colonoscopy), as well as adherence to all screening tests for which participants were age-eligible. Dimensions of religiousness assessed included church participation, religious support, active and passive spiritual health locus of control, and positive and negative religious coping. Results showed that roughly half (46 %) of the sample had not received all of the cancer screening tests for which they were age-eligible. In multivariate analyses, positive religious coping was significantly associated with adherence to all age-appropriate screening (OR = 5.30, *p* < .01). Additional research is warranted to replicate these results in larger, more representative samples and to examine the extent to which enhancement of religious coping could increase the impact of cancer control interventions for Latinas.

## Introduction

Latinos represent the largest and fastest growing ethnic minority population in the United States (U.S. Bureau of the Census 2010). Although the incidence of many cancers is lower among Latinos compared to non-Latino whites, Latino men and women are less likely to survive most cancers, even after accounting for differences in age (American Cancer Society 2009). Lower rates of survival likely reflect diminished access to early detection, diagnostic, and treatment services. Many Latinos face financial, structural, and sociocultural barriers to health care and are therefore less likely to seek and receive healthcare services (American Cancer Society 2008; Balluz et al. 2004). Latinos have lower rates of screening for breast, cervical, and colorectal cancer compared to non-Latino whites (American Cancer Society 2008). Persistent cancer disparities suggest the need for new approaches to reach this population.

Faith-based organizations (FBOs) are a promising avenue to reach Latinos. Ninety percent of Latinos report membership in a religious group (Cadge and Ecklund 2007; Espinoza et al. 2003). Moreover, religious institutions play a prominent role in Latino communities, shaping numerous behaviors from political decisions to daily family life (Pew Hispanic Center and Pew Forum on Religion and Public Life 2007). Qualitative research suggests that Latinas, in particular, view religiousness/spirituality as an important dimension of overall health (Jurkowski et al. 2010).

Religiousness is a multidimensional construct that reflects the shared beliefs and practices of a faith-based, social organization (Miller and Thoresen 2003). A sizeable body of literature documents relationships between religiousness and health outcomes (Chatters et al. 1998; Koenig et al. 2001; Powell et al. 2003). Recent research has focused on the link between different dimensions of religiousness and preventive health behaviors (e.g., Benjamins et al. 2011; Holt et al. 2009a). However, little is known about the specific religious factors that may impact preventive health behaviors among Latinos (Benjamins 2007).

Religious coping theory (Pargament 1997) and social support theory (Israel 1990) suggest several possible avenues through which religious factors may influence use of preventive health services, including cancer screening behaviors. First, *church* *participation*, generally defined as frequency of attendance at religious services or other church-related events (Idler 1999), may influence exposure to church norms (e.g., no smoking and moderation of alcohol). Second, increased access to *religious support*, defined as instrumental, informational, or emotional assistance exchanged within a religious community, may buffer stressful life events, thereby providing increased ability to cope with negative events (e.g., abnormal screening results) (Krause 1999; Pérez et al. 2011; Strawbridge et al. 1997; van Olphen et al. 2003). Third, one’s relationship with a higher power may affect perceived control over health behaviors and outcomes (Thoresen and Harris 2002). For instance, a collaborative relationship with a higher power in the management of one’s health, known as an *active* *spiritual health locus of control,* may empower people to engage in behaviors beneficial for their own health. Alternatively, a *passive spiritual health locus* of control may lead people to rely solely on God to determine their health (Holt et al. 2003, 2007), akin to what has been described as *fatalismo* (fatalism)—a sense that one’s life outcomes are beyond one’s control (Abraído-Lanza et al. 2007; Añez et al. 2005). Finally, religion may impact health behaviors through *religious coping,* a construct that reflects how people utilize religion to understand and deal with stressors (Pargament 1997; Pargament et al. 2000). *Positive religious coping* reflects benevolent religious methods of understanding and managing life stressors, whereas *negative religious coping* reflects religious struggles in coping (Pargament et al. 1998). An association between religious coping and health suggests the presence of stressors in a population. In the current sample of participants, factors such as low socioeconomic status, racism, language barriers, undocumented immigration status, and lack of access to health care were expected to be stressors.

A better understanding of the specific dimensions of religiousness associated with health behaviors (in this case, cancer screening) among Latinas could enable the development of effective, religiously tailored interventions to promote cancer early detection with the ultimate goal of reducing health disparities. Studies conducted with African-American populations suggest that incorporation of religious themes into health interventions may enhance their relevance, improve program participation, and, ultimately, boost intervention efficacy (Campbell et al. 2007; Holt et al. 2009b; Voorhees et al. 1996; Yanek et al. 2001). Church-based interventions have been used to promote cancer education and cancer screening among low-income Latinas (e.g., Duan et al. 2000; Fox et al. 1998b; Lopez and Castro 2006). However, to our knowledge, none of these programs have integrated religious content into their health promotion messages. Therefore, we sought to understand the relationship between varied dimensions of religiousness and cancer screening practices among Latinas in order to inform such interventions. Specifically, we explored the association between cancer screening practices and four dimensions of religiousness: (1) church participation, (2) perceived religious support, (3) spiritual health locus of control, and (4) religious coping.

## Method

### Setting and Sample

We conducted a cross-sectional survey among members of a moderate-sized Baptist church located in Boston, Massachusetts. Data were collected as part of a pilot intervention study to evaluate the feasibility and impact of a peer-led cancer screening intervention that incorporated spiritual themes and practices.

A current church roster was used to identify 97 potentially eligible respondents. Those eligible for participation were (1) 18 years of age or older, (2) self-identified as Hispanic or Latino, and (3) Spanish or English speaking. We included only women in the analysis due to the small number of men in the congregation who were age-eligible for screening ages 50+. To promote participation, the pastor made announcements after worship services during the data collection period, informing congregants about study procedures and purpose. In addition, personalized invitations signed by the pastor and principal investigators were distributed. In-person reminders following church services and up to three telephone calls were made to non-respondents.

Female Spanish-speaking research assistants conducted in-person interviews immediately following religious services in church offices that afforded privacy. Data collection took place between May and October of 2009. Voluntary completion of the survey following the provision of verbal consent information constituted informed consent. The study protocol was approved by the Institutional Review Boards at the University of Massachusetts Boston and the Harvard School of Public Health.

### Measures

Where available, we employed existing validated scales that had been previously tested among Latino populations. To ensure accurate cultural interpretation, we forward- and back-translated data collection tools and subsequently conducted cognitive interviews among 10 Latinos to assess item comprehension, as well as linguistic and cultural appropriateness (Willis 2004). Participants in cognitive interviews were recruited from a demographically similar Spanish-speaking Baptist church *not* participating in the parent study, and these data were not included in subsequent analyses.

### Outcomes

Outcomes of interest were adherence to cancer screening recommendations current at the time of data collection (American Cancer Society 2009). For women aged 40–49, “adherence” to breast cancer screening recommendations was defined as having had a mammogram within the prior 2 years and a clinical breast examination (CBE) within the prior year. For women over age 50, breast cancer screening adherence was defined as having a mammogram and CBE within the prior year. In terms of cervical cancer, women aged 18 and over who had a Pap test within the prior 3 years were considered compliant. Among those aged 50+, having had an annual fecal occult blood test (FOBT), sigmoidoscopy within prior 5 years, or colonoscopy within prior 10 years was deemed compliant for colorectal cancer screening. Participants who met the criteria for adherence to breast, cervical, *and* colorectal cancer screening tests appropriate for their age were categorized as being compliant with “all age-appropriate screening.”

Items to assess screening behaviors were drawn from the Behavioral Risk Factor Surveillance System (BRFSS; Centers for Disease Control and Prevention 2004). The BRFSS is a state-based survey of the civilian, non-institutionalized adult population that is widely used to measure the prevalence of behavioral risk factors. It has been found to be reliable (Cronbach’s alpha coefficients above 0.70) across a variety of populations, including native Spanish speakers (Stein et al. 1993).

#### Church Participation

Two standardized items assessed frequency of attendance at church services and other activities, with categorical responses ranging from “never” to “every day” (Fetzer Institute & National Institute on Aging Working Group 2003).

#### Religious Support (Krause 1999)

Two items assessed perceived positive religious support from members of the church community with response categories on a four-point scale ranging from “none” to “a great deal.” Two items assessed perceived negative religious support on a four-point scale ranging from “never” to “very often.” The internal consistency of the positive religious support scales was good (α = 0.86). However, the internal consistency of the negative religious support scale was poor (α = 0.37) and thus was dropped from the analyses.

#### Spiritual Health Locus of Control (Holt et al. 2007)

This scale assessed the belief that a higher power (e.g., God) has control over one’s health. Three items assessed an *active* spiritual health locus of control, whereby God plays a collaborative role in one’s health. Three items assessed a *passive* spiritual health locus of control, whereby respondents do not take protective health actions because they believe God is in sole control of their health. All items were measured on a four-point scale ranging from “strongly disagree” to “strongly agree.” The internal consistency of the active dimension was good (α = 0.81). After dropping one reverse-coded item (“God and I share responsibility for my health”) with a low item-total correlation, the internal consistency of the two-item passive dimension also was good (α = 0.79).

#### Religious Coping (Pargament 1999)

Two scales were used to measure how people make use of religion to understand and cope with major problems in their lives (Pargament et al. 1998). Three items measured *positive* religious coping (i.e., benevolent religious methods of understanding and managing life stressors) and three items measured *negative* religious coping (i.e., religious struggle in coping). All items were measured on a four-point scale ranging from “not at all” to “a great deal”. The internal consistency of the positive religious coping scale was adequate (α = 0.74). However, the internal consistency of the negative religious coping scale was not acceptable (α = 0.13) and, therefore, was dropped.

#### Socio-demographic Variables

Age, income, education, gender, proficiency with spoken English, and region of origin/ethnicity were measured using standard items from the BRFSS. Items to assess healthcare access, including type of health insurance, were also from the BRFSS (Centers for Disease Control and Prevention 2004).

### Statistical Analyses

First, we used descriptive statistics (e.g., frequencies, proportions, and means) to describe socio-demographic characteristics of the study sample, distributions of church participation, social support, spiritual health locus of control, religious coping, and adherence to breast, cervical, colorectal, and overall cancer screening guidelines. We were unable to present reliable associations between dimensions of religiousness and adherence to cervical cancer screening among women because fewer than 10 women in this sample had *not* been screened (Hosmer and Lemeshow 2000; Peduzzi et al. 1996). As a second step, we examined characteristics of each religious scale by computing an interitem reliability coefficient (Cronbach’s α). As noted, scales with poor interitem reliability (α < 0.60) were not used in any of the analyses; these included negative religious support and negative religious coping. We then used logistic regression to examine bivariate and multivariate relationships among cancer screening adherence (yes/no), demographic variables, and dimensions of religiousness. Variables selected for inclusion in multivariate models were those that demonstrated bivariate associations (*p* < .10) with adherence to screening recommendations. Analyses included all available data using SPSS statistical software, version 19.0 (IBM Corporation, New York, NY).

## Results

### Characteristics of Study Sample

Of the 97 eligible female church members, 78 (80 %) participated in interviews, which took approximately 45 minutes to complete. Socio-demographic characteristics of the sample are depicted in Table 1. Nearly half (49 %) had a household income of less than $30,000, and almost a third (28 %) did not have health insurance. Over a third (36 %) had less than a high school education, and more than half (60 %) reported that they did not speak English at all or did not speak very well. Respondents were predominantly from Central (49 %) or South America (38 %). The mean length of time living in the United States was roughly 13 years.

**Table 1:** Socio-demographic characteristics of the study sample by category (*N* = 78)

Characteristic	*n*	%^a^
Age		
18–39	31	39.7
40–49	17	21.8
50–59	22	28.2
60+	8	10.3
Missing	0	0.0
Annual household incone		
Less than $10,000	13	16.7
$10,000–$29,999	25	32.1
$30,000–$49,999	19	24.4
More than $50,000	4	5.1
Don’t know	15	19.2
Refused or missing	2	2.6
Employment status		
Employed	51	65.3
Unemployed	10	12.8
Homemaker	6	7.6
Retired	3	3.8
Other	6	7.6
Refused or missing	2	2.5
Insurance status		
Insured (private)	17	21.8
Insured (public)	36	46.1
Not insured	22	28.2
Don’t know or missing	3	3.8
Educational level		
No school or only kindergarten	1	1.3
Grades 1 through 8	19	24.4
Some high school	8	10.3
High school or GED	28	35.9
Some college or technical school	16	20.5
College graduate or higher	6	7.7
Missing	0	0.0
English language proficiency		
Very well, well	31	39.7
Not very well, not at all	47	60.3
Missing	0	0.0
Region of origin		
Caribbean	4	5.1
Mexico	3	3.8
Central America	38	48.7
South America	30	38.4
Other	3	3.8
Missing	0	0.0
Marital status		
Married/living as married	44	56.4
Not married/not living as married	34	43.5
Time living in United States		
Years	13.24 (mean)	8.8 (SD)
Missing	1	

^a^Percent of non-missing; columns may sum to greater than 100 % due to rounding

### Dimensions of Religiousness

Descriptive characteristics of dimensions of religiousness are presented in Table 2. Church participation was high, as 94 % of respondents reported that they attended church at least once a week. Respondents also reported high levels of perceived social support from their church (mean score = 3.3 out of a maximum score of 4.0). Most reported that they could count on fellow church members “some” or “a great deal” for help if they were ill (74 %), or for comfort if they had a problem 84 %. Only 6 % of respondents felt that church members often made too many demands on them, and only 3 % of respondents felt that others in their church were often critical of what they did (results not shown). On average, church members in our sample reported very high levels of active spiritual locus of control (mean score = 3.7 out of a maximum score of 4.0) as well as high levels of positive religious coping (mean score = 3.7 out of a maximum score of 4.0).

**Table 2:** Characteristics of religiousness measures among women (*N* = 78)

Constructs	Cronbach’s α	Mean (SD)
*Church participation*		
How often do you usually attend religious services?	N/A	%^a^
Daily or once per week		93.8 %
Few times per month		4.7
Less than once per year or never		1.6
Besides regular services, how often do you take part in other activities at your place of worship?		
Daily or once per week		48.4 %
Few times per month		26.6
Few times per year		4.7
Less than once per year or never		20.3
*Religious support (positive)*		
1. If you were ill, how much would the people in your congregation help you out? 2. If you had a problem or were faced with a difficult situation, how much comfort would the people in your congregation be willing to give you?	0.87	3.34 (0.91)
*Spiritual health locus of control (active)*		
Please indicate how much you agree with the following statements:	0.86	3.65 (0.60)
1. Living the way the Lord says I’m supposed to means I have to take care of myself. 2. Even though I trust God will take care of me, I still need to take care of myself. 3. God gives me the strength to take care of myself.		
*Spiritual health locus of control (passive)*		
Please indicate how much you agree with the following statements: 1. It’s ok not to seek medical attention because I feel that God will heal me. 2. There is no point in taking care of myself when it’s all up to God anyway.	0.78	1.90 (0.80)
*Religious coping* (*positive*)		
Think about how you try to understand and deal with major problems in your life. To what extent is each of the following involved in the way you cope? 1. I think about how my life is part of a larger spiritual force. 2. I work together with God as partners. 3. I look to God for strength, support, and guidance in crisis.	0.83	3.68 (0.53)

^a^Percent of non-missing; columns may sum to greater than 100 % due to rounding

### Adherence to Cancer Screening Recommendations

Distributions for adherence to cancer screening recommendations are presented in Table 3. Among women between the ages of 40–49, 65 % had a mammogram within the past 2 years. Among women 50 years and older, 60 % had a mammogram within the prior year. Only 60 % of women over 40 had a clinical breast examination within the prior year. Among all women, only 43 % had both screening examinations at recommended intervals. Two-thirds (67 %) of women aged 18 and older reported having had a Pap test within the past year; 88 % have had a Pap test within the prior 3 years. Nearly two-thirds (60 %) of women aged 50 and older were compliant with colorectal cancer screening recommendations. Still, only 54 % of respondents reported having had all the recommended examinations for their age.

**Table 3:** Adherence to screening guidelines

Screening type	*n*	(%)^a^
Colorectal cancer screening (age 50+)	30	
Compliant with recommendations^b^	18	(60)
Mammography screening (age 40–49)	17	
Within past 1–2 years	11	(65)
More than 2 years ago	2	(12)
Never	4	(24)
Mammography screening (age 50+)	30	
Within past year	18	(60)
Within past 2 years	7	(23)
More than 2 years ago	2	(7)
Never	3	(10)
Clinical breast examination (age 40+)	47	
Within past year	28	(60)
More than 1 year ago	6	(13)
Never	11	(23)
Don’t know/not sure	2	(4)
All breast cancer screening recommended for age^c^	47	
Yes	20	(43)
No	27	(57)
Cervical cancer screening^d^ (age 18+)	58	
Within past year	39	(67)
Within past 3 years	12	(21)
More than 3 years ago	1	(2)
Never	6	(10)
Adherence to all screening tests recommended for age^e^	78	
Yes	42	(54)
No	36	(46)

^a^Columns may not sum to 100 % due to rounding; non-answers were coded as “No” or “Never”

^b^Annual FOBT, sigmoidoscopy every 5 years, or colonoscopy every 10 years

^c^For women aged 40–49: mammogram within prior 2 years and clinical breast examination within prior year; for women aged 50+: mammogram and clinical breast examination within prior year

^d^Pap smear within prior 3 years

^e^For cervical cancer screening, 20 cases were missing due to reported hysterectomy. For those 20 cases, not having the cervical cancer screening was *not* considered non-adherence in calculating adherence to all screening tests

### Associations with Cancer Screening

In bivariate analyses, age (OR = 0.92; 95 % CI = 0.88–0.96), English language proficiency (OR = 2.60; 95 % CI = 1.00–6.70), passive spiritual health locus of control (OR = 0.52; 95 % CI = 0.28–0.96), and positive religious coping were significantly associated with all age-appropriate screening (*p* < .10). None of the variables were significantly associated with adherence to individual screening tests (data not shown).

Variables with significant bivariate relationships were entered into a multivariate logistic regression model (Table 4). In this model (*p* < .05), age, positive religious coping, and passive spiritual health locus of control remained significantly associated with all age-eligible screening, but English language proficiency was no longer significant. In a final model (with English language proficiency removed), positive religious coping remained strongly associated (OR = 5.30; 95 % CI = 1.18–23.71), while passive spiritual health locus of control was reduced to marginal significance (OR = 0.50; 95 % CI = 0.24–1.00).

**Table 4:** Multivariate results for adherence to cancer screening recommendations

All cancer screening (*N* = 78)		
Correlates	OR	95 % CI
*Model 1* (*Nagelkerke R* ^*2*^ = *.40; % correctly classified* = *72* *%*)		
Age	0.91***	0.86–0.96
English language proficiency	0.69	0.18–2.66
Spiritual health locus of control (passive)	0.48*	0.24–0.99
Religious coping (positive)	5.51*	1.20–25.25
*Final model* (*Nagelkerke R* ^*2*^ = *.40; % correctly classified* = *73* *%*)		
Age	0.92***	0.88–0.96
Spiritual health locus of control (passive)	0.50~	0.24–1.00
Religious coping (positive)	5.30*	1.18–23.71

All logistic regressions were conducted using the enter method. Variables selected for inclusion in multivariate models were those that demonstrated significant bivariate associations with adherence to screening recommendations (*p* < .10). Results are not presented for breast, cervical cancer, or colorectal cancer screening individually due to small cell sizes

~ *p* < .10, * *p* < .05, ** *p* < .01, *** *p* < .001

## Discussion

We sought to identify possible relationships between dimensions of religiousness—church participation, religious support, spiritual health locus of control, and religious coping—and adherence to cancer screening recommendations. The most noteworthy finding was a strong association between positive religious coping and adherence to all age-appropriate screening, even after controlling for relevant covariates. For every one-point increase on the positive religious coping scale, the odds of having completed all cancer screenings were increased by a factor of 5.3. Positive religious coping involves actively seeking spiritual support and working in partnership with God to solve problems (Pargament 1999). Such actions may reduce anxiety about getting screened or receiving positive test result. Cancer screening may be perceived as a “risky” behavior among some Latinos; one risks discovering that one has cancer through screening, which is often linked to *muerte* (death) in the Latino community (Fernandez et al. 2008). In this context, positive religious coping may help individuals overcome fear that may act as a barrier to screening.

While passive spiritual locus of control was negatively associated with all age-appropriate cancer screening, it did not achieve statistical significance. Notably, few in our sample expressed a highly passive spiritual health locus of control. Levels of active spiritual locus of control—a collaborative relationship with God—were much higher. This finding is consistent with qualitative work by Flórez et al. (2009), showing that breast cancer screening behaviors among Dominican women were influenced by a combination of internal (personal agency) and external (e.g., based on God’s will) forces. It was also consistent with qualitative work among healthy Latina women, showing that an active relationship with God in maintaining one’s health was much more common than a passive relationship where God alone is responsible for one’s health (Jurkowski et al. 2010). Taken together, these findings suggest that simplistic conceptions of *fatalismo* may not accurately reflect the predominant health locus of control about cancer screening and prevention among Latinas (Abraído-Lanza et al. 2007).

Overall, we found low rates of adherence to all screening tests for which one was eligible; nearly half of respondents needed one or more of the recommended examinations. However, adherence to individual cancer screening tests in our predominantly female sample was similar to Massachusetts screening rates among Hispanic women (Massachusetts Department of Public Health 2009). For example, 88 % of women aged 18 and older in our sample reported having had a Pap test within the past 3 years, compared to 86 % in a Massachusetts sample (Massachusetts Department of Public Health 2009). Mammography in the past 2 years for women aged 50+ was 83 % in our sample, compared to 89 % for women aged 40+ in Massachusetts (Massachusetts Department of Public Health 2009).

Not surprisingly, we found high levels of church participation among our church-based sample; the majority of respondents were at the church on a daily or weekly basis. Although respondents frequently attended church and reported high levels of positive religious support, neither of these variables was associated with cancer screening practices. Prior studies of these relationships have been mixed, with some finding no association (Fox et al. 1998a; Katz et al. 2008) and others finding positive associations (Benjamins 2006) between church attendance, self-rated religiousness, and breast cancer screening. In our sample, there was very little variability in these measures (i.e., ceiling effects), which may partially account for the lack of associations.

Before discussing implications, there are a number of study limitations that must be noted. First, data were collected from a small, purposive sample of low-income, Spanish-speaking Latinas primarily from Central and South America. Although the focus on an underserved, minority sample is a strength of the study, generalizability of this data is limited. Second, participants self-reported both cancer screening behaviors and religiousness, which leaves open the possibility of recall and social desirability biases. To minimize the potential for this, we assessed the religious measures after the cancer screening items to minimize order effects. Third, the negative religious coping and negative religious support variables had very low interitem reliability and, therefore, could not be included in the analyses. This may be due to a floor effect, as very few participants in this highly religious sample endorsed these items. Finally, we used a cross-sectional design, which limits our ability to explore temporal or cause-effect relationships. Accordingly, relationships between religiousness and screening utilization may be explained by additional unknown, unmeasured factors. For example, an underlying personality trait, such as optimism, could have influenced both religious coping and cancer screening behaviors (Koenig, et al. 2001).

Nevertheless, we believe this to be one of the few studies to employ multidimensional measures of religious/spiritual constructs that assess distal aspects of religion (e.g., church attendance) and proximal aspects of religion (e.g., religious coping) that may influence cancer screening behaviors in a Latina population. If proximal religious predictors of cancer screening behaviors can be identified, they should be examined as potential mechanisms in church-based interventions. Understanding these underlying mechanisms could enhance the efficacy of cancer control interventions.

Churches provide access to a large segment of the Latino population—including people of diverse ages, socioeconomic levels, and ethnic groups (Pew Hispanic Center & Pew Forum on Religion and Public Life 2007), and provide existing infrastructures, communication networks, and facilities that are useful for health promotion activities. As such, they represent a promising venue for the dissemination of health interventions. Understanding the aspects of religion that can support health behaviors will be helpful in designing interventions that resonate with a church-based audience. If replicated in a larger, more representative sample, our findings suggest that religious coping can play an important role in motivating cancer screening. Such investigations are warranted given the health disparities among Latinos.
